# Toll-like receptor signalling via IRAK4 affects epithelial integrity and tightness through regulation of junctional tension

**DOI:** 10.1242/dev.201893

**Published:** 2023-12-11

**Authors:** Jesse Peterson, Kinga Balogh Sivars, Ambra Bianco, Katja Röper

**Affiliations:** 1 MRC-Laboratory of Molecular Biology, Francis Crick Avenue, Cambridge Biomedical Campus, Cambridge CB2 0QH, UK; 2 Oncology R&D, Precision Medicine and Biosamples, R&D, AstraZeneca, Pepparedsleden 1, Nova, Mölndal, SE-431 83, Sweden; 3 Clinical Pharmacology and Safety Sciences CPSS Oncology Safety, AstraZeneca, Darwin Building, Cambridge Science Park, Milton Road, Cambridge CB4 0WG, UK

**Keywords:** Toll-like receptors, IRAK4, Intestinal epithelium, Epithelial barrier, Tight junctions, Cytoskeleton, Tension

## Abstract

Toll-like receptors (TLRs) in mammalian systems are well known for their role in innate immunity. In addition, TLRs also fulfil crucial functions outside immunity, including the dorsoventral patterning function of the original Toll receptor in *Drosophila* and neurogenesis in mice. Recent discoveries in flies suggested key roles for TLRs in epithelial cells in patterning of junctional cytoskeletal activity. Here, we address the function of TLRs and the downstream key signal transduction component IRAK4 in human epithelial cells. Using differentiated human Caco-2 cells as a model for the intestinal epithelium, we show that these cells exhibit baseline TLR signalling, as revealed by p-IRAK4, and that blocking IRAK4 function leads to a loss of epithelial tightness involving key changes at tight and adherens junctions, such as a loss of epithelial tension and changes in junctional actomyosin. Changes upon IRAK-4 inhibition are conserved in human bronchial epithelial cells. Knockdown of IRAK4 and certain TLRs phenocopies the inhibitor treatment. These data suggest a model whereby TLR receptors near epithelial junctions might be involved in a continuous sensing of the epithelial state to promote epithelial tightness and integrity.

## Introduction

Epithelial tissues are one of the major tissue types in all animals and provide the building blocks for most organs in both invertebrates and vertebrates. Furthermore, organ morphogenesis during development in many cases commences from initially simple epithelial primordia. Epithelia also represent the first barrier of defence against infection and help mount an innate immune response.

Toll-like receptors (TLRs) are key components of the innate immune response, with their activation through pathogen-related molecules leading to transcription and production of inflammatory modulators, e.g. cytokines (Leulier and Lemaitre, 2008; Netea et al., 2012). Most TLRs are single-pass transmembrane receptors with a leucine-rich extracellular region (LRR) that recognises pathogen-associated molecular patterns (PAMPs) on bacteria, viruses, fungi, and other unicellular pathogens. This pathway is conserved from invertebrates to mammals. The activated receptor recruits to its intracellular tail the adaptor MyD88, which through its death domain binds to the downstream kinases IRAK4 (interleukin-1 receptor associated kinase 4) and then IRAK1 or IRAK2 (interleukin-1 receptor associated kinase 1/2) to form the so-called myddosome. Through further downstream components, this eventually leads to degradation of cytoplasmic IκB (inhibitor of nuclear factor κ-B), releasing NFκB (nuclear factor κ-light-chain-enhancer of activated B cells) to activate targets in the nucleus (Netea et al., 2012). NFκB-dependent gene transcription regulates a variety of targets including factors that are directly involved in the immune response, such as cytokines and chemokines, but also cell adhesion molecules, growth factors and their receptors and apoptosis related factors (http://www.bu.edu/nf-kb/gene-resources/target-genes/). TLR signalling has been implicated in a significant number of immune disorders, and therefore members of this pathway, such as TLRs, IRAK kinase family members and inhibitory κB kinases (IKKs), have come to attention as promising drug targets. Not all components of the pathway are essential, with redundancy built into the system. Mutations in some of the genes involved in TLR signalling, such as IRAK4, are compatible with life in both human and mouse in the absence of infection; however, in individuals carrying the mutations the innate immune response is reduced and sometimes not effective (Picard et al., 2010). Individuals with IRAK4 mutations, for example, show an increased sensitivity to infections, specifically in the upper respiratory tract (Ku et al., 2007). Other TLR mutations, including those TLRs analysed here (TLR1, -2, -4, -6), have been linked to a broad spectrum of immune disorders and chronic infections, such as colitis in mice and common variable immunodeficiency, asthma, Crohn’s disease, atherosclerosis, tuberculosis and leprosy in humans (Choteau et al., 2017; Lin et al., 2012; Mortaz et al., 2017). The absence of lethality for many of the mutations suggests redundancy in the system, and, in particular, IRAK1 has been suggested to be able to compensate for lack of IRAK4. Indeed, during a Phase I clinical trial one of the inhibitors used in this study (PF-06650833) revealed no evidence of a severely disrupted epithelial barrier (Danto et al., 2019). With regards to IRAK4 inhibition and knockdown, we suspect that the acute effects we observe in either case are masked in the developmental context by such redundancy, hence leading to overall viability (Bennett et al., 2023; Qin et al., 2004; Rosenbaum et al., 2022).

TLRs were first identified in *Drosophila* where, similar to mammals, several TLRs exist. In the fly, where their role has been extensively investigated, important functions during innate immune responses, but also during development, have emerged. In fact, the receptor Toll itself was initially characterised during the process of dorsoventral axis formation in the fly embryo, during which localised receptor activation leads to the nuclear localisation of one of the fly NFκBs, Dorsal, in only the future ventrally located cells (Gerttula et al., 1988). Further roles for other Tolls in *Drosophila* have been elucidated more recently, including functions during wound healing, tube morphogenesis, as well as during axis elongation (Carvalho et al., 2014; Kolesnikov and Beckendorf, 2007; Lavalou et al., 2021; Pare et al., 2014; Tamada et al., 2021), with each of these roles impinging on actomyosin activity. The role in wound healing requires the standard downstream cascade, including MyD88 as well as the downstream IRAK kinases Tube and Pelle (fly IRAK4), whereas their requirement during tube formation and axis elongation remains unresolved.

The role of Toll receptors during axis extension, termed germband extension in *Drosophila*, deserves particular attention. Here, these receptors, which in the early fly embryo are expressed in a striped pattern downstream of the anterior-posterior patterning machinery, localise to apical junctions in the epithelial cells, and mis-expression studies show that they can drive accumulation of actomyosin at the level of adherens junctions. Furthermore, this role appears to depend on heterophilic Toll–Toll receptor interactions, which were confirmed in a heterotypic expression system (Pare et al., 2014) and also through a heterophilic interaction with a G protein-coupled receptor (Lavalou et al., 2021). The potential for TLR–TLR interaction has already been suggested for the original Toll (Keith and Gay, 1990), but is thus far better characterised in flies than in mammals (Anthoney et al., 2018; Ward et al., 2015). Many family members of the wider LRR-type group engage in homophilic and heterophilic interactions (Özkan et al., 2013), supporting the notion that such interactions could be a key part of TLR function. Such function would also place TLRs in a common group with other homophilic/heterophilic cell surface receptors, such as cadherins, nectins and Crumbs: their levels and ability to engage in trans with receptors on neighbouring cells have important roles in patterning actomyosin activity within the apical-junctional region of epithelial cells (Chang et al., 2011; Thompson et al., 2013).

Junctional patterning of actomyosin activity plays key roles during morphogenesis of epithelial tissues, allowing for defined changes in the shape of the apical domain of epithelial cells (Harris, 2018). Furthermore, junctional tension due to actomyosin contractility is clearly important for the assembly and maintenance of both adherens junctions and tight junctions (Citi, 2019; Itoh et al., 2012; Terry et al., 2011).

As type I transmembrane proteins, the biosynthesis of TLRs involves trafficking to the correct cellular membrane destination. Fly Tolls, where this has been analysed, appear to be localised near apical junctions in the embryonic epidermis (Pare et al., 2014; Towb et al., 1998). The steady-state distribution for mammalian TLRs depending on the TLR type varies between plasma membrane and endosomal localisation, where this has been analysed (Tan and Kagan, 2016). Similar to flies, a number of human TLRs are expressed in epithelial tissues (http://www.proteinatlas.org) and some of these human TLRs show a polarised distribution to either apical or basolateral plasma membrane domains in the lung (Ioannidis et al., 2013) and gut (Price et al., 2018). This polarisation, at least for innate immunity-related functions, most likely reflects an adaptation to where the recognised target PAMPs are most likely to be encountered.

While TLRs and signalling through IRAK4 have been linked to alterations in epithelial barrier function in the presence of bacteria (Al-Sadi et al., 2021; Guo et al., 2013; Kuo et al., 2013; Ragupathy et al., 2014; Ruffner et al., 2019), little is known about whether they share a physiological role with fly Tolls in establishing and maintaining tissue integrity. Here, we set out to uncover whether TLRs and downstream components play a non-immune role in mammalian epithelial cells, using differentiated human Caco-2 cells as an epithelial intestinal model system and primary airway epithelial cells grown in an air–liquid interface culture as a model of lung tissue. Caco-2 cells show a polarised and junctional localisation of a subset of TLRs. Using inhibitors of the downstream pathway kinase IRAK4 as well as RNA interference against pathway components including TLRs, we show that at steady state a baseline level of pathway activation is detected, and that absence of this signalling leads to disruption at the tight junctions, including loss of junctional tension and a strong reduction in trans-epithelial electrical resistance and, thus, epithelial barrier function. We suggest that baseline TLR signalling, possibly downstream of TLR–TLR interaction between neighbouring cells in the apical junctional domain, serves a homeostatic role in re-enforcing junctional tension through actomyosin at tight junctions, thereby promoting epithelial tightness.

## Results

### TLRs are localised to apical domain and junctions in differentiated Caco-2 cells concomitant with steady-state pathway activation

**Fig. 1:**
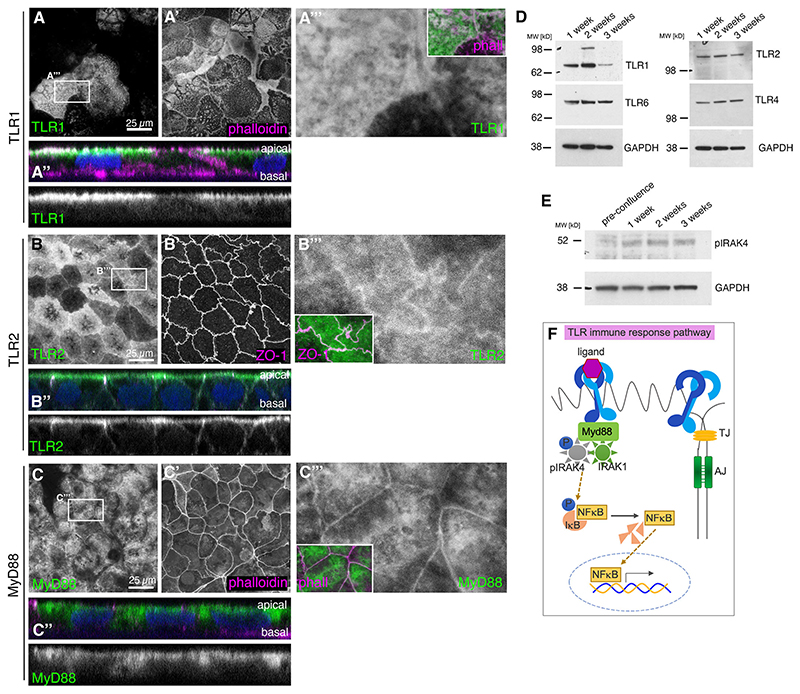
TLRs are constitutively present and localised in a polarised position in epithelial Caco-2 cells. (A-C‴) Localisation of TLR1 (A-A‴), TLR2 (B-B‴) and the downstream scaffold protein MyD88 (C-C‴) in epithelial Caco-2 cells at 3 weeks post-confluence. z-projections of apical-most confocal sections are shown. Antibody labelling against TLR1, TLR2 and MyD88 is in green; phalloidin and ZO-1 in magenta are used to label cell boundaries. A″-C″ show an apical-basal cross-section of the epithelium (nuclei stained with DAPI in blue); apical is up, individual channels for TLR1, TLR2 and MyD88 are shown below. A‴-C‴show magnifications of the channels for TLR1, TLR2 or MyD88, respectively; the insets show the overlay. (D) TLR1, TLR2, TLR4 and TLR 6 are all detected in confluent Caco-2 cells during maturation over 3 weeks by western blotting. Note that TLR1 levels progressively decrease in fully matured cells. (E) Levels of phospho-IRAK4 (pIRAK4) increase with maturation of Caco-2 cells in culture, indicating steady-state pathway activation and increased signalling with more mature junctions. (F) In innate immunity signalling, TLR binding of an immuno-active ligand triggers assembly of the myddosome containing pIRAK4, IRAK1 and MyD88, leading to IκB phosphorylation and dissociation from NFκB, which is now free to enter the nucleus and affect transcription. A constitutive, non-immune role of the TLR pathway would not require a ligand but might go through the same pathway. See also Fig. S1. AJ, adherens junction; TJ, tight junction.

Caco-2 cells grown on Transwell filters differentiate and mature into highly polarised enterocyte-like cells, with well-defined junctional regions, dense apical microvilli and well-separated apical and basolateral domains. At 1-3 weeks post-confluence, TLR1, TLR2, TLR4 and TLR6, all TLRs previously described to localise to the plasma membrane, were all found to be expressed in Caco-2 cells, and all localised to the apical plasma membrane and overlapped with the apical-junctional regions, with some, e.g. TLR2, displaying junctional enrichment in cross-sections (Fig. 1A-B‴, Fig. S1). Similarly, the MyD88 adaptor protein, which links the TLRs at the plasma membrane to the downstream IRAKs (Fig. 1F), was also localised apically and junctionally (Fig. 1C-C‴). Biochemical analysis revealed that TLR2, TLR4 and TLR6 levels remained constant during the time in culture, whereas TLR1 levels dropped with differentiation and maturation (Fig. 1D).

The presence of TLRs on the epithelial surface, in the absence of any infection, appeared to be able to trigger activation of the downstream pathway (Fig. 1F), as we could detect phosphorylated IRAK4 (p-IRAK4) in these cells, and p-IRAK4 levels increased with the cells reaching confluence and differentiating (Fig. 1E). The presence of low-level p-IRAK4 is consistent with findings in other lysates of kidney and immune cell lines (Vollmer et al., 2017). This baseline TLR signalling via p-IRAK4 did not seem to involve nuclear translocation of NFκB, as we did not detect NFκB in the nucleus in control Caco-2 monolayers, in contrast to the positive control (interleukin 1b-treated) (Fig. S1C-F).

Thus, intestinal epithelial Caco-2 cells showed a robust and polarised expression of TLRs as well as a baseline activation of the TLR pathway in the absence of infection and, thus, absence of immune triggers of the pathway.

### IRAK4 inhibition leads to reversible loss of epithelial integrity at tight junctions

**Fig. 2:**
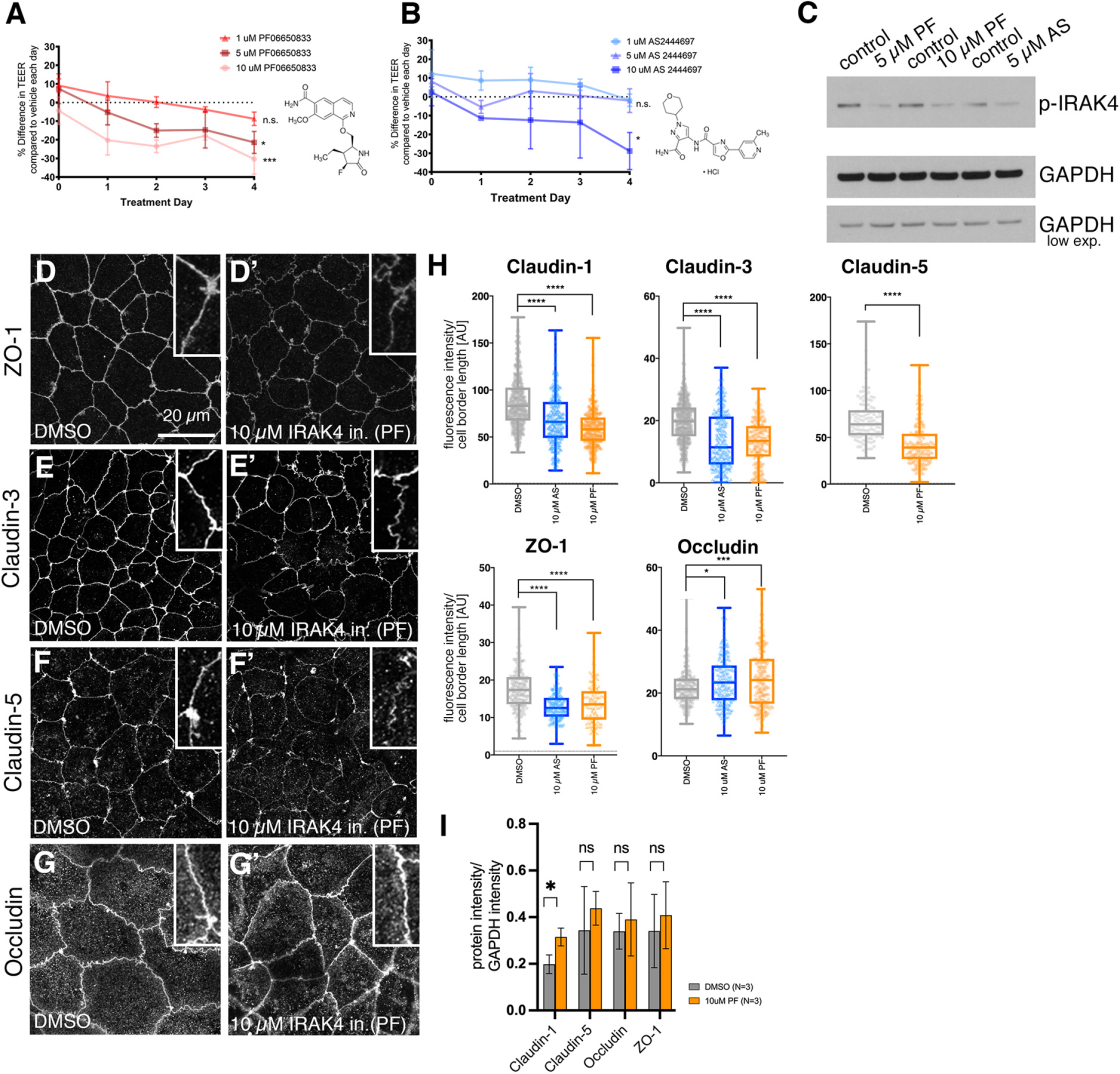
IRAK4 inhibition affects tight junction integrity and barrier function. (A,B) Treatment of 3-week post-confluent Caco-2 cell monolayers with IRAK4 inhibitor, either PF-06650888 (A; PF) or AS2444697 (B; AS), induces a dose-dependent reduction in TEER. Chemical structures of each inhibitor are indicated. Shown are mean±s.e.m. of *n*=6 (DMSO), 6 (AS), 4 (PF) separate Transwells. Statistical significance at the 4 day time point was determined by one-way ANOVA with Tukey’s multiple comparison test. Note that both AS and PF are compared with same DMSO controls, run contemporaneously. *P<0.05; ***P<0.005. n.s., not significant. Chemical structures of the inhibitors are shown. (C) Treatment with 5 μM and 10 μM PF or 5 μM AS leads to a reduction in p-IRAK4. GAPDH was used as a loading control; standard and low exposure are shown. (D-H) Effect of PF and AS treatment on junctional components after 4 days of treatment. z-projections of confocal sections covering the apical-lateral junctional area are shown for cells treated with PF (D-G′), with quantification for both PF- and AS-treated cells (H). ZO-1 (D,D′), claudin 3 (E,E′) and claudin 5 (F,F′) localisation at cell–cell junctions is reduced upon IRAK4 inhibition with 10 μM PF, whereas occludin is slightly increased (G,G′). (H) Quantification of fluorescence intensity changes at cell–cell junctions in control (DMSO), 10 μM AS- and 10 μM PF-treated Caco-2 epithelial cell layers. Total junctions analysed from three images comprising one representative image per Transwell for three separate Transwells were: claudin 1: 547 for DMSO, 413 for 10 μM AS, 368 for 10 μM PF; claudin 3: 602 for DMSO, 358 for 10 μM AS, 211 for 10 μM PF; claudin 5: 120 for DMSO, 192 for 10 μM PF; ZO-1: 194 for DMSO, 127 for 10 μM AS, 142 for 10 μM PF; occludin: 221 for DMSO, 262 for 10 μM AS, 204 for 10 μM PF. Box-and-whisker plots in this and all subsequent figures show mean, 25th and 75th percentile, with extreme data points indicated by whiskers. Statistical significance was determined by unpaired Student’s *t*-test or one-way ANOVA with Dunnett’s multiple comparison test when comparing two or more drug treatments (****P<0.00001; ***P<0.0001; *P<0.01). (I) Quantification of protein levels of tight junction components from triplicate blots for control and 10 μM PF-treated Caco-2 cells (4 days treatment). Significance was determined by unpaired Student’s *t*-test (*P=0.0214). ns, not significant. Data are shown as mean±s.e.m. See also Fig. S2. AU, arbitrary units.

In order to test whether TLR pathway activation leading to IRAK4 phosphorylation is important for epithelial function, we blocked IRAK4 function using two commercially available inhibitors of IRAK4, PF-06650833 (hereafter PF; Lee et al., 2017) and AS244697 (hereafter AS; Kondo et al., 2014) in a range from 1 to 10 μM (Fig. 2A,B). TLR signalling had previously been linked to regulation of epithelial integrity and particularly tightness, although always under infection paradigms (Clarke et al., 2011; Kuo et al., 2013; Ragupathy et al., 2014; Ruffner et al., 2019). We therefore decided to focus initially on the effect of IRAK4 inhibition on epithelial barrier function and tightness.

Epithelial monolayers of Caco-2 cells treated with either inhibitor showed a dose-dependent decrease in trans-epithelial electrical resistance (TEER), a measure of tight junction integrity and epithelial tightness, in a reversible fashion (Fig. 2A,B, Fig. S2A,B). IRAK-inhibitor treatment also led to greater trans-epithelial diffusion (Fig. S2C). The inhibition was targeting IRAK4, as levels of p-IRAK4 decreased in a dose-dependent manner (Fig. 2C) whereas total IRAK4 remained constant (Fig. S2H). We analysed various components of tight junctions after 4 days of treatment in order to assess which aspect of barrier function had been compromised by the IRAK4 inhibition. The cytoplasmic adaptor of tight junctions ZO-1 (TJP1) showed a marked decrease in localisation to tight junctions upon IRAK4 inhibition with either AS or PF (Fig. 2D,D′,H; reduction of 27.2% and 22.1%, respectively), whereas intensity of the transmembrane protein occludin slightly increased (Fig. 2G-H; increase of 10.1% and 13.7%, respectively). Claudins form another family of tight junction proteins, characterised by four transmembrane domains. Claudin 1, 3 and 5 all showed a marked decrease at tight junctions upon inhibitor treatment [Fig. 2E-F′,H;Fig. S2D,D′; reductions of 20.5% (AS)and 30.0% (PF) for claudin 1, 42.2% (AS) and 31.8% (PF) for claudin 3 and 38.3% (PF) for claudin 5]. E-cadherin (cadherin 1) remained localised to adherens junctions, but the extent of lateral spot adherens junctions basal to the adherens junction belt seemed reduced (Fig. S2E-G). The changes at tight and adherens junctions appeared to be largely caused by relocalisation of proteins away from the junctions rather than degradation and loss of protein, as at the total protein level most components remained at a steady level (Fig. 2I, Fig. S2I,J).

These data indicate that loss of the p-IRAK4 signal causes a reduction of epithelial barrier function in Caco-2 monolayers, suggesting that the above observed baseline activation of the pathway at steady state could serve to reinforce the barrier, even in the absence of an immune response.

### Knockdown of IRAK4 leads to loss of epithelial integrity at tight junctions

**Fig. 3:**
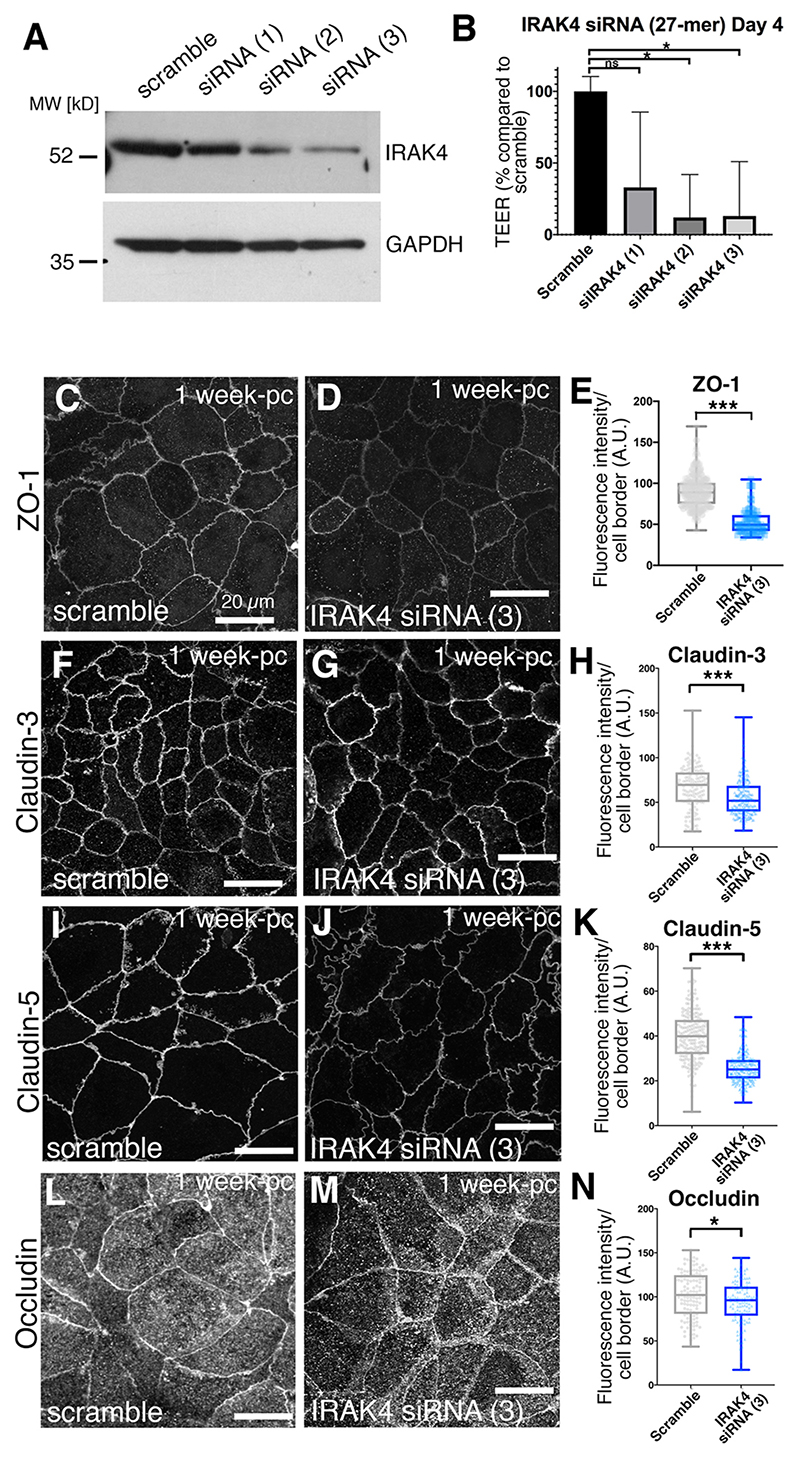
TLR interference phenocopies IRAK4 inhibition and reduction. (A) siRNA treatment of 1-week post-confluence Caco-2 monolayers with three different siRNAs targeting IRAK4 compared with a scrambled control leads to various levels of reduction in IRAK4 levels. GAPDH was used as loading control. (B) In siRNA-treated cells with reduced IRAK4 levels (siRNAs 2 and 3), TEER at day 4 of siRNA treatment is strongly reduced compared with the scrambled control. *n*=3 Transwells were analysed. Shown are mean+s.d. Significance was determined by one-way ANOVA and Dunnett’s multiple comparison test. *P<0.05. ns, not significant. (C-E) In Caco-2 monolayers treated with siRNA (3) against IRAK4, ZO-1 levels at junctions are reduced by 40.2% compared with scramble control. *n*=250 junctions were analysed for the scramble control and *n*=209 for IRAK4 siRNA (3). Statistical significance was determined using unpaired *t*-test (***P<0.0001). (F-H) In Caco-2 monolayers treated with siRNA (3) against IRAK4, claudin 3 levels at junctions are reduced by 15.5% compared with scramble control. *n*=141 junctions were analysed for the scramble control and *n*=258 for IRAK4 siRNA (3). Statistical significance was determined using unpaired *t*-test (***P<0.0001). (I-K) In Caco-2 monolayers treated with siRNA (3) against IRAK4, claudin 5 levels at junctions are reduced by 34.7% compared with scramble control. *n*=202 junctions were analysed for the scramble control and *n*=227 for IRAK4 siRNA (3). Statistical significance was determined using unpaired *t*-test (***P<0.0001). (L-N) In Caco-2 monolayers treated with siRNA (3) against IRAK4, occludin levels at junctions are slightly reduced by 8.2% compared with scramble control. *n*=111 junctions were analysed for the scramble control and *n*=110 for IRAK4 siRNA (3). Statistical significance was determined using unpaired Student’s *t*-test (*P=0.0147). In C,D,F,G,I,J,L,M, *z*-projections of confocal sections covering the apical-lateral junctional area are shown. A.U., arbitrary units; pc, post-confluence.

In order to confirm the effects observed under IRAK4 inhibition, we decided to also reduce IRAK4 levels by small interfering RNA (siRNA). As 3-week post-confluent Caco-2 monolayers could not efficiently be transfected, we established a regime of siRNA treatment at confluent seeding and compared epithelial tightness and junctional composition at 1-week post-seeding when the control monolayer had reached a minimum resistance of 600 Ω/cm^2^. Caco-2 cell monolayers treated with siRNA directed against IRAK4 showed a marked decrease in total IRAK4 (Fig. 3A). The monolayers also displayed a consistent and statistically significant reduction in TEER (Fig. 3B). When we analysed tight junction components, ZO-1 showed a clear reduction (40.2%) in fluorescence intensity at cell boundaries (Fig. 3C-E), similar to what was observed when Caco-2 monolayers were treated with either AS or PF inhibitor. Furthermore, in line with our observations under inhibitor treatment, claudin 3 and claudin 5 were reduced at junctions (Fig. 3F-H for claudin 3, 15.5% reduction; Fig. 3I-K for claudin 5, 34.7% reduction). Occludin, by contrast, was slightly reduced in its membrane localisation under IRAK4-siRNA (Fig. 3L-N, 8.2% reduction). This is possibly because reducing IRAK4 levels would reduce p-IRAK4 even more than the inhibition, or because, due to the nature of the siRNA experiment, the age of the monolayers was not identical between chemical inhibition and siRNA treatment as discussed above.

These results overall confirm the findings obtained with chemical inhibition of IRAK4 described above and support the idea that steady-state TLR signalling via p-IRAK4 in epithelial monolayers plays a role in the reinforcement of epithelial barrier tightness. Treatment with IRAK4 inhibitor affected monolayers more homogeneously than siRNA and could be applied to more mature monolayers. We therefore performed our further analyses using both chemical inhibition of IRAK4 as well as siRNA-induced reduction in protein levels, as these complement each other in terms of efficacy and time point of treatment.

### Loss of epithelial integrity downstream of IRAK4 inhibition is due to loss of junctional tension

**Fig. 4:**
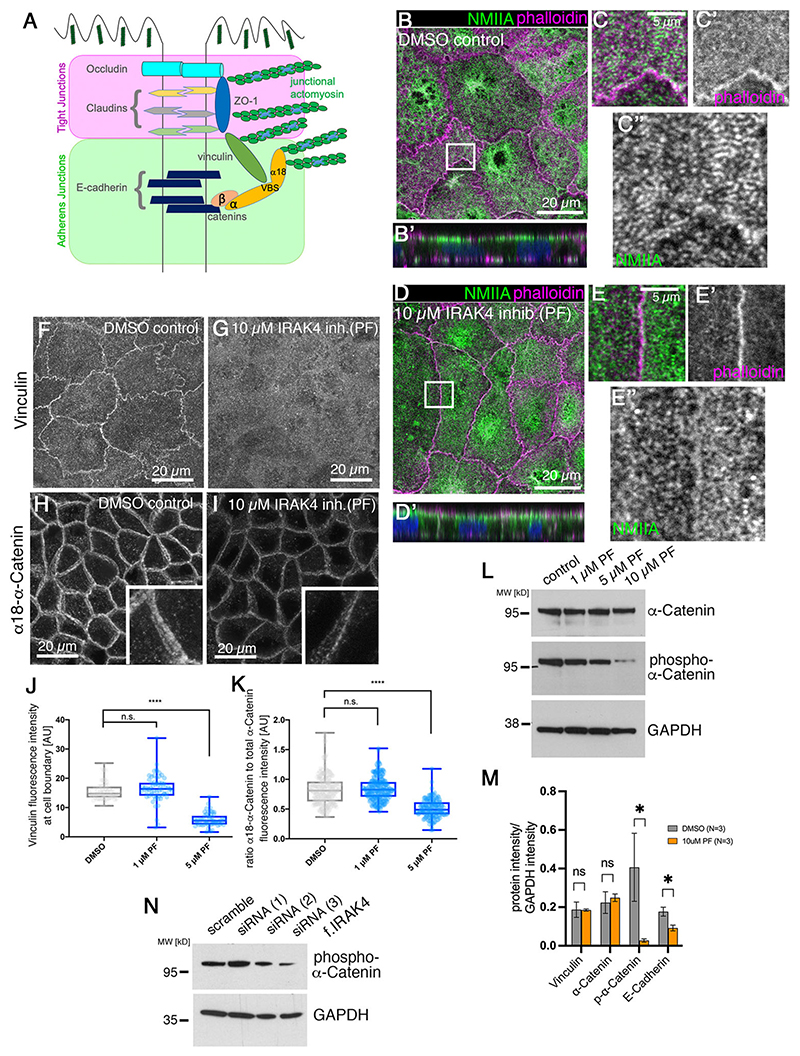
IRAK4 inhibition leads to loss of epithelial tension at tight junctions. (A) Schematic illustrating junctional transmembrane and cytoplasmic components of adherens and tight junctions, showing the transmembrane components occludin, claudins and E-cadherin, and the cytoplasmic linkers ZO-1, vinculin and catenins that mediate the link to actin and mechanosensitivity. (B-E″) Apical NMIIA in differentiated Caco-2 cell monolayers shows an intricate striated pattern across the free apical surface (B,C″) and along cell–cell junctions (B,C,C″), whereas upon 10 μM PF treatment (D-E″) NMIIA apical localisation is much more diffuse und unstructured (D,E,E″). NMIIA is in green and phalloidin to label F-actin is in magenta (B-E). *z*-projections of confocal sections covering the apical surface and junctional area are shown (B′,D′). Boxed areas in B and D are shown at higher magnification in C-C″ and E-E″. NMIIA channels only of panels B and D are shown in Fig. S3. (F-I) The mechanosensitive cytoplasmic junctional proteins vinculin (F) and α-catenin (H) are reduced upon treatment with 10 μM PF (G,I). α-Catenin (H,I) is revealed using an antibody against the open and thus stretched conformation of α-catenin (α18). *z*-projections of confocal sections covering the apical-lateral junctional area are shown. Insets show higher magnifications of the junctional area of an individual cell. (J,K) Quantification of changes to junctional vinculin (J) and α-catenin (K) fluorescence intensity in control and IRAK4 inhibitor (PF)-treated differentiated Caco-2 cells. α-Catenin intensity is expressed as the ratio between α-catenin under tension revealed by α18 and total α-catenin. For vinculin, *n*=62 junctions were analysed for the scramble control, *n*=63 for 1 μM PF treatment and *n*=52 for 5 μM PF treatment. For α-catenin/α18–α-catenin, *n*=196 junctions were analysed for the scramble control, *n*=203 for 1 μM PF treatment and *n*=143 for 5 μM PF treatment. Statistical significance was determined using one-way ANOVA with Dunnett’s multiple comparison test (****P<0.00001). n.s., not significant. AU, arbitrary units. (L) Serine-phosphorylation of α-catenin compared with total α-catenin is reduced in a dose-dependent manner with PF treatment in Caco-2 cell monolayers. GAPDH was used as loading control. (M) Quantification of protein levels of the indicated junctional components from triplicate blots for control and 10 μM PF-treated Caco-2 cells. Data are shown as mean±s.e.m. Significance was determined by unpaired Student’s *t*-test (*P=0.0204 for p-α-catenin; *P=0.0057 for E-cadherin). ns, not significant. (N) siRNA treatment against IRAK4 of Caco-2 cell monolayers also leads to a reduction in phospho-α-catenin; shown is treatment with the same three siRNAs as shown in Fig. 3. See also Figs S3-S5.

A key aspect of the establishment and maintenance of tight junctions is tension exerted onto these junctions by actomyosin activity (Fig. 4A) (Itoh et al., 2012; Miyake et al., 2006; Terry et al., 2011). We therefore analysed non-muscle myosin IIA (NMIIA) as well as junctional components involved in anchoring of actomyosin to junctions under control and IRAK4-inhibitor treatment conditions. NMIIA in the apical and junctional region of Caco-2 cells at 3-weeks post-confluence was organised into striated patterns, suggesting a mini-sarcomere-like arrangement of actomyosin within this region (Fig. 4B-C″, Fig. S3A-A″). Under IRAK4 inhibitor treatment (PF), NMIIA was less organised (Fig. 4D-E″, Fig. S3B-B″), with foci of myosin across the apical surface as well as near junctions, but many fewer clear striations (Fig. 4E-E″, Fig. S3B-B″). NMIIA labelling also extended further basally along the lateral junctions compared with the control, where the highest intensity of NMIIA labelling was confined to the apical and apical-junctional region (compare Fig. 4B′ and D′).

Both adherens junctions and tight junctions are linked to and regulated by the junctional actomyosin cytoskeleton (Citi, 2019). The cytoplasmic adaptor ZO-1 binds to claudins and occludin but also contains an actin-binding domain (Fig. 4A) (Furuse et al., 1994; Itoh et al., 1999a,b). Furthermore, ZO-1 has also been shown to be able to bind directly to α-catenin (Maiers et al., 2013), and α-catenin in this capacity can influence tight junction assembly and integrity. α-Catenin is also a key mechanosensitive component of adherens junctions (Huveneers and de Rooij, 2013). A further component in contact with both adherens and tight junctions is the protein vinculin, which also acts as a mechano-sensor, requiring activation and interaction with actin filaments to unfold (Bakolitsa et al., 2004; Janssen et al., 2006). We therefore used antibodies directed against vinculin and against total α-catenin as well as an antibody (α18) directed against the form of α-catenin that is under tension and unfolded, and thereby able to bind vinculin (Yonemura et al., 2010), and that we use therefore as an indicator of junctional tension. Vinculin in control (DMSO-treated) cells colocalised with apical junctions (Fig. 4F), whereas upon treatment with IRAK4 inhibitor (PF) the protein was diffuse and not concentrated at apical junctions (Fig. 4G,J), although protein levels did not appear to be affected (Fig. S3H). When analysing α-catenin, while total α-catenin intensity at junctions was slightly reduced upon IRAK4 inhibition (Fig. S4E), α18 labelling at junctions was further reduced as a proportion of total α-catenin, suggesting a loss of tension (Fig. 4H,I,K, Fig. S3C-D‴,F,G), and such reduction was also observed when targeting IRAK4 by siRNA (Fig. S3I-M). Only the stretched form of α-catenin that is recognised by the α18 antibody can be bound by vinculin (Seddiki et al., 2018); therefore, the change in α18–α-catenin observed could be upstream of the observed change in vinculin. Recent data show that phospho-regulation of α-catenin in a flexible linker region is another pathway of regulating intercellular adhesion (Escobar et al., 2015). Interestingly, levels of phosphorylated α-catenin (detecting phosphorylation at S655 and T658) decreased in a dose-dependent manner with increasing amounts of IRAK4-inhibitor used (Fig. 4L, M) and were also reduced when IRAK4 levels were reduced using siRNA (Fig. 4N). This suggests that the TLR-IRAK4 pathway could in fact impinge on α-catenin as a target.

In our standard protocol, changes upon IRAK4 inhibition were assessed after 4 days of treatment, but we wanted to investigate which, if any, changes we could observe after 1 day of treatment with IRAK4 inhibitor (Fig. S4). ZO-1 did not change intensity at junctions after 1 day of treatment, and there was only a slight reduction in E-cadherin. By contrast, the amount of both phospho-α-catenin and α18–α-catenin compared with total α-catenin did decrease (75% and 53%, respectively; Fig. S4C-E), suggesting that α-catenin might be the initial target of IRAK-4 signalling.

Thus, inhibition of IRAK4 appears to lead to a reduction in actomyosin-generated tension at tight junctions. Because such tension has previously been shown to be crucial to maintain junctional integrity (Citi, 2019; Itoh et al., 2012; Terry et al., 2011), the loss of epithelial tightness upon IRAK4 inhibition could be a secondary effect of the loss of apical actomyosin tension across the epithelial layer.

### IRAK4 signalling in junction integrity is conserved in human primary respiratory bronchial epithelial cells

In order to address whether signalling from IRAK4 regulates epithelial tightness through junctional tension across epithelia and not only in Caco-2 cells, we turned to human primary bronchial epithelial cells cultured on filters in an air–liquid interface culture. These differentiated respiratory cells presented well-established adherens and tight junctions, as well as a network of junctional and apical actomyosin surrounding patches of apical motile cilia (Fig. S5A-B′). Similar to Caco-2 cells, the respiratory cells showed a dose-dependent reversible decrease in TEER when treated with the inhibitor PF targeting IRAK4 (Fig. S5C). When treated with 10 μM IRAK4 inhibitor these cells showed a reduction in junctional ZO-1, claudin 3, E-cadherin and vinculin, as well as NMIIA and levels of phospho-α-catenin and α18–α-catenin compared with total α-catenin (Fig. S5D-K).

These results indicate that the regulation of epithelial tension and tightness downstream of baseline IRAK4 signalling is not restricted to colon epithelial cells but might be a conserved epithelial feature.

### IRAK4 signalling in junction integrity relays a TLR signal

In order to assess whether the effects on epithelial tightness and junctional organisation due to IRAK4 activity were downstream of the apical junctional TLRs, we used siRNA directed against a subset of TLRs to study the downstream effects in Caco-2 monolayers.

**Fig. 5:**
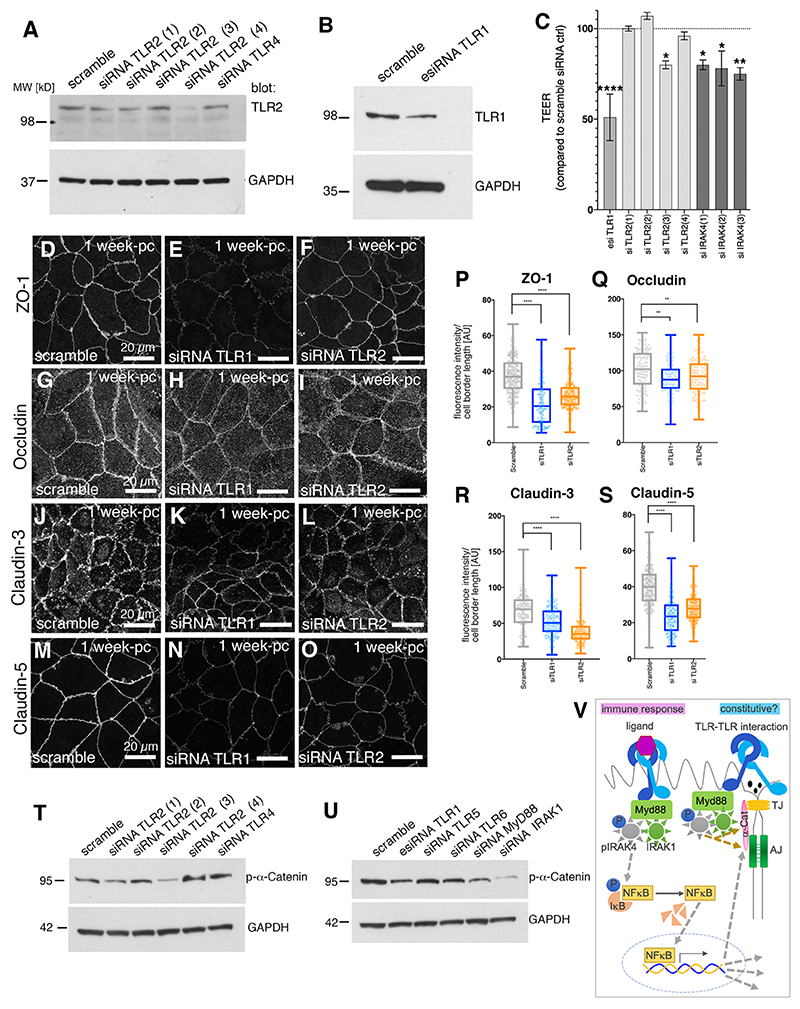
Reduction of TLR levels leads to loss of epithelial tightness and changes at junctions. (A,B) Western blot analysis of protein levels in 1-week post-confluence Caco-2 monolayers upon treatment with siRNAs targeting TLR2 (1-4) or TLR4 as control (A) as well as TLR1 (B), to demonstrate a reduction in protein level for TLR2 and TLR1. GAPDH was used as loading control. (C) siRNA treatment of Caco-2 monolayers with different siRNAs targeting TLR1, TLR2 or IRAK4 compared with a scramble control leads to reduction in TEER level. *n*=5 independent Transwells (scramble, siTLR1, siTLR2, siIRAK4) were averaged. Data are shown as mean ±s.e.m. Statistical significance was determined by one-way ANOVA with Dunnett’s multiple comparison test (*P<0.01; **P<0.001; ****P<0.00001). (D-O) In siRNA-treated 1-week post-confluence (pc) Caco-2 monolayers with reduced TLR1 or TLR2 levels, localisation of junctional components to cell borders is affected. *z*-projections of confocal sections covering the apical-lateral junctional area are shown: (D-F) ZO-1, (G-I) occludin, (J-L) claudin 3 and (M-O) claudin 5. (P-S) Quantification of components in scramble control, TLR1 siRNA- and TLR2 siRNA-treated Caco-2 cells. (P) Knockdown of TLR1and TLR2 leads to ZO-1 reduction at junctions of 44.5% and 30.0%, respectively. *n*=267 junctions were analysed for the scramble control, *n*=160 for siTLR1 and *n*=216 for siTLR2. (Q) Knockdown of TLR1and TLR2 leads to mild occludin reduction at junctions of 14.3% and 9.7%, respectively. *n*=111 junctions were analysed for the scramble control, *n*=125 for siTLR1 and *n*=93 for siTLR2. (R) Knockdown of TLR1and TLR2 leads to reduction of claudin 3 at junctions of 27.3% and 49.2%, respectively. *n*=141 junctions were analysed for the scramble control, *n*=175 for siTLR1 and *n*=142 for siTLR2. (S) Knockdown of TLR1and TLR2 leads to reduction of claudin 5 at junctions of 41.2% and 30.3%, respectively. *n*=202 junctions were analysed for the scramble control, *n*=168 for siTLR1 and *n*=187 for siTLR2. Statistical significance was determined using one-way ANOVA with Dunnett’s multiple comparison test as **P<0.001, ****P<0.00001. Scramble values for claudin 5 and occludin are the same as those in Fig. 3 as these were run contemporaneously. (T) Analysis of phospho-α-catenin levels upon treatment with siRNA against TLR2 (1-4) or against TLR4 compared with scramble control. GAPDH is shown as loading control. (U) Analysis of phospho-α-catenin levels upon treatment with siRNA against TLR1, TLR5, TLR6, MyD88 or IRAK1 compared with scramble control. GAPDH is shown as loading control. (V) Model of ‘constitutive’ TLR pathway function compared with the immune response-induced pathway. In addition to the pathway described in immune responses converging on IRAK4 (left), our data suggest a possible constitutive pathway of TLR signaling through IRAK4 that affects junctional components and the cytoskeleton through α-catenin. See also Fig. S6. AJ, adherens junction; TJ, tight junction.

When levels of TLR1 or TLR2 were reduced using different sets of siRNAs (Fig. 5A,B), we observed a significant reduction in TEER (Fig. 5C, Fig. S6). Conversely, treatment with a TLR1/2 agonist (CU-T12-9; Cheng et al., 2015) using a regime identical to the siRNA treatment timeline, led to a significant increase in TEER compared with control (Fig. S6J). We then analysed junctional components when TLR1 or TLR2 (siRNA3) were reduced using siRNA. ZO-1, occludin, claudin 3 and claudin 5 were reduced at cell borders under either treatment [Fig. 5D-S; ZO-1 reduction: 40.9% (siTLR1), 28.9% (siTLR2); occludin reduction: 11.5% (siTLR1), 9.9% (siTLR2); claudin 3 reduction: 19.9% (siTLR1), 41.6% (siTLR2); claudin 5 reduction: 40.5% (siTLR1), 28.6% (siTLR2)]. This was comparable to what we observed when IRAK4 was reduced by siRNA and under inhibitor treatment. Furthermore, we again observed changes indicative of a reduction in junctional tension when TLR1 and TLR2 were reduced. At 1 week post-confluence, actomyosin structures across the apical surface of Caco-2 cells were not yet as elaborated into sarcomere-like assemblies across the apical region as at 3 weeks post-confluence, although they could already be observed at junctions (Fig. S6A,A′; compare with Fig. 4B-C′). When TLR1 or TLR2 were knocked down by siRNA, similar to IRAK4 knockdown by siRNA (Fig. S6A-E), junctional myosin II intensity was reduced, as was its junctional organisation into striated patterns (Fig. S6A′-D′). In addition, siRNA treatments against TLR1, TLR2, MyD88 and IRAK1 all reduced levels of p-α-catenin in the cells to some extent (Fig. 5T,U), again suggesting that the changes observed at tight junctions when the TLR-IRAK4 pathway was inhibited or impaired were downstream of changes in junctional tension.

In summary, the above data support a model whereby TLR1 and TLR2 are involved in sensing of the epithelial state and, through downstream MyD88, IRAK4 and IRAK1, could help to mature and maintain junctional tension and, hence, epithelial tightness.

## Discussion

TLRs constitute a large family of membrane receptors conserved through evolution that fulfil many diverse functions both during development and throughout life. While this family includes both cell-surface and endosomal receptors known to respond to different stimuli, the majority of TLR signalling is reported to converge on a common signalling hub, the myddosome, and information is relayed from here to the nucleus. Recent data from *Drosophila*, however, suggests functions that might involve the myddosome but do not effect changes through altering transcription. In agreement with this, our data suggest that a common epithelial role of the upstream part of the pathway might impinge on junctional integrity, and hence epithelial barrier function, through the modulation of junctional tension.

A link between TLR signalling and epithelial barrier function via IRAK4 signalling has been well described during infection progression, although stimulation of different TLRs depending on the tissue context can lead to either a decrease or an increase in barrier function. For instance, recent studies suggest that during infection of the lung some bacteria could exploit a transient relaxation of the epithelial barrier downstream of TLR signalling (though through the non-canonical downstream components p38/MAPK) to invade the host (Clarke et al., 2011). This increase in leakiness of the epithelium, characterised by a decrease in expression of the tight junction-associated claudins, as well as an increase in SNAI1 expression (a transcriptional repressor of claudins), could allow egress of immune cells as well as antimicrobial factors into the lung lumen as part of the host defence. Conversely, differential TLR-mediated control of junction strength in the Peyer’s patches of gut epithelium enhances leakiness to allow localised sampling of the luminal surface by dendritic cells as part of normal immune surveillance (Davies et al., 2010). Interestingly, recent studies of neurobehavioral deficits in *Tlr2* knockout mice indicated problems at junctions (Hu et al., 2020). *Tlr2* knockout mice are viable but display a range of brain-related problems, in agreement with TLR2 being expressed widely in glial cells and neurons in the nervous system (Hayward and Lee, 2014). These mice at 12 months of age displayed blood–brain barrier problems, concomitant with a reduction of protein levels of ZO-1, occludin and claudin 5 in this tissue. Several studies of murine gut inflammation support a key role for TLR1 and TLR2 in maintaining barrier integrity. *Tlr1* knockout mice exhibit decreased proliferation rates in the colonic crypt and impaired recovery of the tissue after colitis induction (Kamdar et al., 2018). TLR2 stimulation had a protective effect on tight junctions in animals, explants and primary human intestinal cells in culture, and both *Tlr2* and *Myd88* knockout mice exhibit an accelerated disruption of the barrier following colitis induction (Cario et al., 2007). Additionally, a recent study demonstrated that *Tlr1* and *Tlr2* (but not *Tlr6*) knockout mice display increased intestinal permeability and pathogenic yeast colonisation in a colitis model (Choteau et al., 2017).

How essential or redundant are epithelial barrier function of the TLR pathway and its components? As mentioned above, not all mutations in the pathway are lethal, as there is clear redundancy in the system, with TLRs being partially able to compensate for one another and IRAK1 being able to compensate for IRAK4 function (Bennett et al., 2023; Qin et al., 2004; Rosenbaum et al., 2022). Indeed, during a Phase I clinical trial no evidence of a severely disrupted epithelial barrier was detected upon administration of one of the inhibitors used in this study (PF) (Danto et al., 2019). With regards to IRAK4 inhibition and knockdown, we suspect that the acute effects we observe are masked in the developmental context by such redundancy, hence leading to overall viability.

Although this work suggests a role for TLRs through IRAK4 in the maintenance of epithelial barrier function, the precise mechanism of TLR/IRAK4-mediated control of junctional components requires further investigation. There is no known IRAK4 consensus site on α-catenin, and recent evidence suggests that the kinase activity of IRAK4 is dispensable for the function of the myddosome (De Nardo et al., 2018). Furthermore, it is not yet clear how IRAK1, the major downstream effector of IRAK4, is involved in the epithelial barrier function, although IRAK1 was recently identified in an affinity biotinylation screen of E-cadherin binding partners (Guo et al., 2014).

A TLR/IRAK4-mediated complex formation that maintains α-catenin phosphorylation may explain the observed decrease in TEER, which is thought to be primarily a measure of the ‘pore’ pathway mediated by claudins. α-Catenin is a mechanosensitive link between the circumferential adherens junction and the apicolateral tight junction, and its phosphorylation helps maintain intercellular adhesion in both human cell culture and *Drosophila* (Escobar et al., 2015). In addition, by maintaining the ‘unfolded’ form of α-catenin to allow recruitment of vinculin, TLR/IRAK4 signalling may help buffer the tight junctions from disruption by mechanical forces (Konishi et al., 2019). Importantly, despite our observations of altered junctional proteins, we do not observe large-scale changes in total protein levels under IRAK4 inhibition or siRNA, and, following removal of IRAK4 inhibitors, TEER rapidly recovered to control levels. This suggests a translocation of proteins away from the cell–cell interface rather than protein degradation, although the precise mechanism remains to be determined.

How does the modulation of junctional tension that we observe when either IRAK4 or TLR1/2 are targeted fit with other developmental data? In *Drosophila*, one key function for TLRs has emerged during the early morphogenetic process of germband elongation that occurs during gastrulation (Pare et al., 2014). A combinatorial effect of heterophilic TLR–TLR interaction at boundaries of striped expression domains of different TLRs in the embryonic epidermis leads to actomyosin accumulation at such boundaries. Different TLRs were shown to be able to interact heterophilically in a heterologous expression system. We suspect that, in the case of epithelial monolayers such as the one analysed here, homo- or heterophilic interactions of TLRs localised to the apical-junctional region relay a steady-state signal to the pathway (Fig. 5V). In Caco-2 cells, our data show, this signal leads to the strengthening of actomyosin interactions with junctions and involves the mechanosensitive component α-catenin and its binding partner vinculin. It will be interesting to address in the future whether the effect of TLRs and IRAK4 on junctional myosin in *Drosophila* involves the same set of effectors.

## Materials and Methods

### Cell culture, transfection and inhibitor treatment

Caco-2 cells were grown on 24- or 6-well Transwell polycarbonate inserts with 0.4 μm pore size (Greiner Bio-One) at a density of 10^5^ cells/cm^2^. Complete growth medium consisted of DMEM/Glutamax (Gibco, 10566016) supplemented with 10% fetal bovine serum (Gibco), 1% penicillin/streptomycin, and 1× MEM non-essential amino acids (Gibco) and was changed every other day. Unless otherwise indicated, cells were grown for 21 days post-confluence to achieve full apicobasal polarisation and junction maturation. For transfection studies, cells were reverse-transfected with Lipofectamine 2000 (Invitrogen) with 100 ng of plasmid on a 24-well Transwell insert in 200 μl antibiotic-free media as directed by the manufacturer. For drug studies, IRAK4 inhibitors (PF-06650833 or AS2444697) were applied as DMSO suspensions to both apical and basal chambers at concentrations of 1, 5 and 10 μM, and changed every day for four consecutive days, similar to concentrations used in other studies (Deliz-Aguirre et al., 2021; Li et al., 2019; Matsuoka et al., 2020; Pletinckx et al., 2020). For controls, DMSO was added to media at a concentration equal to the amount of DMSO in the highest concentration of drug tested.

Human bronchial epithelia (NHBE) cells were obtained from Lonza. Cells were expanded in Pneumacult EX media (STEMCELL Technologies) until ready for differentiation, at which point they were grown to confluence on 24-well Transwell inserts with 0.4 μm pore size (Greiner Bio-One). Upon reaching confluence, apical media was removed and the basal media was replaced with Pneumacult ALI media (STEMCELL Technologies). Cells were differentiated for 2 weeks prior to TEER measurement. Drug concentration and dosage regiment were the same as for Caco-2 cells, although drug was only added to basal media.

### TEER measurements

TEER measurements were carried out using an EVOM^2^ Voltometer (World Precision Instruments) with STX2 chopstick electrodes. Both samples and calibration media were allowed to acclimatise to room temperature prior to measurements to minimise fluctuations in resistance during cooling. To accommodate for the air–liquid interface when growing NHBEs, basal media was removed, and fresh base media containing drug was added to the apical and basal compartments. After measurement, the apical media was removed and the samples returned to culture. Resistance was calculated using Ohm’s law and is reported as Ωcm^2^. In some instances, data were normalised to matched controls to improve clarity, as noted in figure legends. Prior to any treatments, samples below a TEER cut-off of 600 Ωcm^2^ were excluded from the analysis.

For the graphs presented in Fig. 2, values shown represent means from three independent experiments (*N*=3) and are expressed as percentage change from vehicle controls. For each experiment, means from three to six wells per condition were normalised to vehicle controls to allow for differences in baseline resistance based on daily temperature and monolayer age. Error bars indicate s.e.m. Statistical significance for the final time point (day 4) was determined by one-way ANOVA with Dunnett’s multiple comparison test.

### FITC-Dextran permeability assay

Caco-2 cells at 2 weeks post-confluence were treated with PF for 4 days as indicated above. After 4 days, Transwells were gently washed once with PBS and transferred to a new 24-well plate containing PBS in the basal compartment. Then, 50 μM FITC-Dextran (FD4, Sigma-Aldrich) in PBS was added to the apical chamber and the cultures were incubated for 2 h. Following incubation, Transwells were removed and FITC diffusion to the basal compartment was read on a Tecan Spark plate reader.

### Immunofluorescence and imaging

Cells were washed briefly in PBS and fixed in 4% methanol-free paraformaldehyde (2% for anti-claudin antibodies) for 15 min at room temperature. They were then washed three times and permeabilised in PBS containing 0.1% saponin for 10 min, followed by blocking in PBS containing for 0.1% saponin and 1% bovine serum albumin for 1 h. Transwell membranes were then excised from plastic supports with a scalpel, and incubated with primary antibodies (see Table S1) overnight at 4°C in blocking buffer. Membranes were then washed three times with PBS and incubated with species-matched Alexa Fluor-conjugated secondary antibodies and phalloidin in blocking buffer, washed three times in PBS, followed by counterstaining with DAPI and mounting using Vectashield (Vector Laboratories). For all TLR antibodies and MyD88, signal was enhanced using a Tyramide Superboost kit (Invitrogen). Cells were imaged on a Leica SP8 or Olympus FluoView 1200. Images were processed using Fiji software to adjust colour palette, contrast and balance; all changes were applied to the entire image and for comparisons the same settings were applied to all images being compared. *z*-projections of confocal sections (standard deviation projections) either covering the apical and apical-junctional area or the whole lateral side were analysed (all fluorescence panels shown in figures are standard deviation projections). Fluorescence intensity was quantified using Fiji. Briefly, junctions were traced using the freehand tool with a line thickness matched to the thickest junction within the set of images to be compared (usually 10-15 pt). Average signal intensity over junction length or width was measured. For each image, background intensity was sampled and averaged from four or five cytoplasmic regions approximately the length of a junction and subtracted from all values within the image. For NMIIA images, ratios were determined by dividing junction intensities by intracellular NMIIA intensity, as determined by averaging three or four circular regions of interest covering intracellular spaces.

### Constructs for transfection

Fluorescent TLR6-YFP was obtained from Addgene (pcDNA3-TLR6-YFP, deposited by Doug Golenbock; plasmid #13020).

### Biochemistry and western blotting

Samples for western blot analysis were grown on 6-well Transwell polycarbonate inserts with 0.4 μm pore size (Greiner Bio-One) at a density of 10^5^ cells/cm^2^. All subsequent steps were conducted on ice or at 4°C. For lysis, membranes were washed twice with ice-cold PBS containing cOmplete™ protease inhibitor and phosSTOP™ phosphatase inhibitor cocktails (Roche). Cells were then gently lifted from membranes with cell scrapers (Corning) and centrifuged at 1000 rpm (200 ***g***) for 5 min. Supernatant was removed and cells were resuspended in lysis buffer containing 20 mM Tris pH 7.5, 1 mM EDTA pH 8.0, 1% Triton X-100, 150 mM NaCl and protease/phosphatase inhibitors (Roche). Samples were passed through a 29 g syringe and placed on ice for 1 h, vortexing for 30 s every 15 min. Samples were then spun at 10,000 rpm (10,000 ***g***) for 10 min and the pellet was discarded. A BCA protein assay (Thermo Fisher Scientific) was used to determine concentration on a NanoDrop 2000 (Thermo Fisher Scientific). Samples were then equilibrated to 2 μg/μl with lysis buffer, 1× LDS sample Buffer (Invitrogen) and 1 mM dithiothreitol. Then, 40 μg of protein were added to each lane and samples were electrophoresed on 4-12% Bis-Tris gels (NuPage, Thermo Fisher Scientific). Gels were then transferred to PVDF membrane, blocked in blocking buffer consisting of 0.1% Tween 20 and 5% milk (Marvel) in PBS. Membranes were then probed overnight with primary antibodies (see Table S1) in blocking buffer at 4°C. The following day, membranes were washed three times with blocking buffer and incubated for 1 h in horseradish peroxidase-conjugated secondary antibody. Membranes were then washed twice with blocking buffer for 1 h total, washed briefly with PBST (PBS plus 0.1% Tween 20), and incubated with Prime ECL (Invitrogen) for 10 min before imaging.

Western blots were quantified by densitometry using Fiji as described (http://www.yorku.ca/yisheng/Internal/Protocols/ImageJ.pdf). Briefly, mean grey values for protein bands, loading controls and background regions were measured and inverted. Backgrounds were then removed from protein bands and loading controls. Protein band/loading control ratios were then averaged for three independent Transwells and compared by unpaired Student’s *t*-test.

### siRNA treatments

The protocol was adapted from a method published on the Bio-Rad website (https://www.bio-rad.com/webroot/web/pdf/lsr/literature/bulletin_5370.pdf). Briefly, Transwells were seeded at high density (10^6^ cells/cm^2^) and reverse transfected with 80 nM siRNA using Lipofectamine 2000. After 6 h, media was replaced with fresh antibiotic-free media to remove excess cells. Overnight confluency was confirmed visually, and after 1 week knockdown efficacy was confirmed by western blot. siRNAs were selected based on efficacy and included standard siRNAs (TLR2: Dharmacon, LQ-005120-01-0005) as well as 27-mer (IRAK4: Origene SR322049) and esiRNAs (Sigma Mission, TLR1: EHU117701, TLR4: EHU086621, TLR6: EHU022071, IRAK1: EHU093291, Myd88: EHU029771).

### Statistical analysis

Unpaired Student’s *t*-test was used for single comparisons between normal distributions, and one- or two-way ANOVA with Dunnett’s or Tukey’s method were used for multiple means comparison unless indicated in text. For all statistical measures, the number of images or wells used to generate data are indicated in figure legends. For fluorescence quantification, replicates were taken from at least three separate membranes stained and imaged contemporaneously, and *z*-stacks were collapsed into a standard deviation projection prior to measurement. Graphs were produced and analysed in Prism. Error bars indicate s.e.m.

All box-and-whisker plots show mean, 25th and 75th percentile, with extreme data points indicated by whiskers. Sample sizes and statistical tests used are indicated in the relevant figure legends.

## Supplementary Material

Supplementary information

## Data Availability

All relevant data can be found within the article and its supplementary information.
