# Supplementary material for: Toll-like receptor signalling via IRAK4 affects epithelial integrity and tightness through regulation of junctional tension
Source: Development. Author manuscript; Available in PMC 2024 Jan 3. (PMC10753582; doi:10.1242/dev.201893)
Supplement: Supplementary information [file EMS192829-supplement-Supplementary_information.pdf]

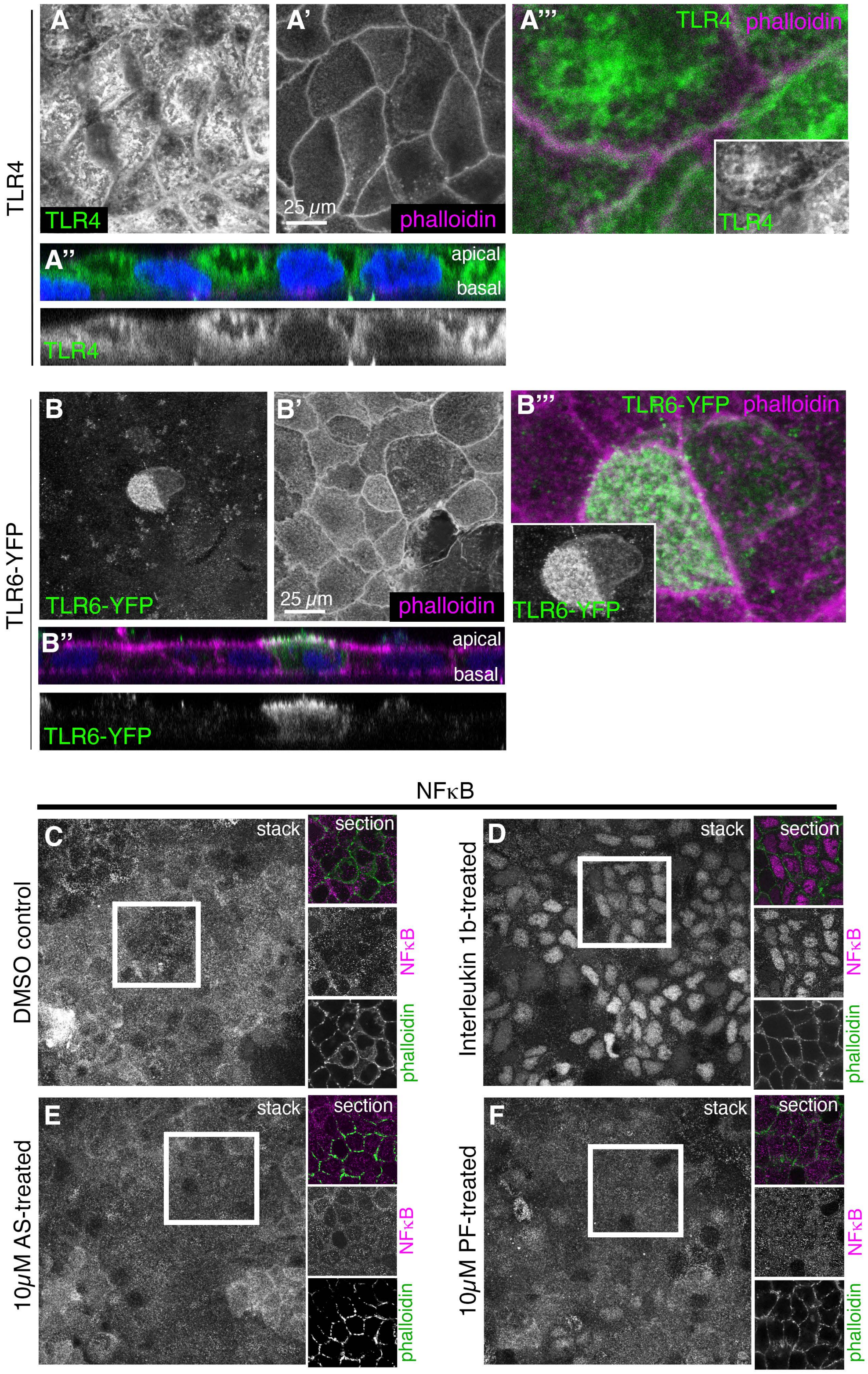

Supplemental Figure S1. Peterson et al.

Fig. S1, related to Figure 1. TLRs are constitutively present and localised in a polarised position in epithelial Caco-2 cells.

Localisation of TLR4 (**A-A’’’**), TLR6-YFP (**B-B’’’**) in epithelial Caco-2 cells. Z-projections of apical-most confocal sections are shown. Antibody labelling against TLR4 and overexpressed TLR6-YFP are in green, phalloidin to label cell boundaries is in magenta. **A’’**, **B’’** show an apical-basal cross-section of the epithelium (nuclei stained with DAPI in blue), apical is up, individual channels for TLR4 and TLR6-YFP are shown below. **A’’’**, **B’’’** show magnifications of cell boundary regions, the insets show the single channel for TLR4 or TLR6-YFP, respectively.

C-F Constitutive TLR signaling in Caco-2 monolayers does not involve nuclear NFκB. 3-week post-confluence Caco2 monolayers have little to no nuclear NFκB (**C**) whereas monolayers treated with 10μM Interleukin-1b, a canonical TLR pathway agonist, show strong nuclear NFκB labeling after 30 mins (**D**). Caco2 monolayers treated with IRAK4-inhibitor (see Fig. 2) like the DMSO control show no nuclear NFκB (**E**, **F**). Cell outlines are labelled with phalloidin revealing F-actin.

Development • Supplementary information

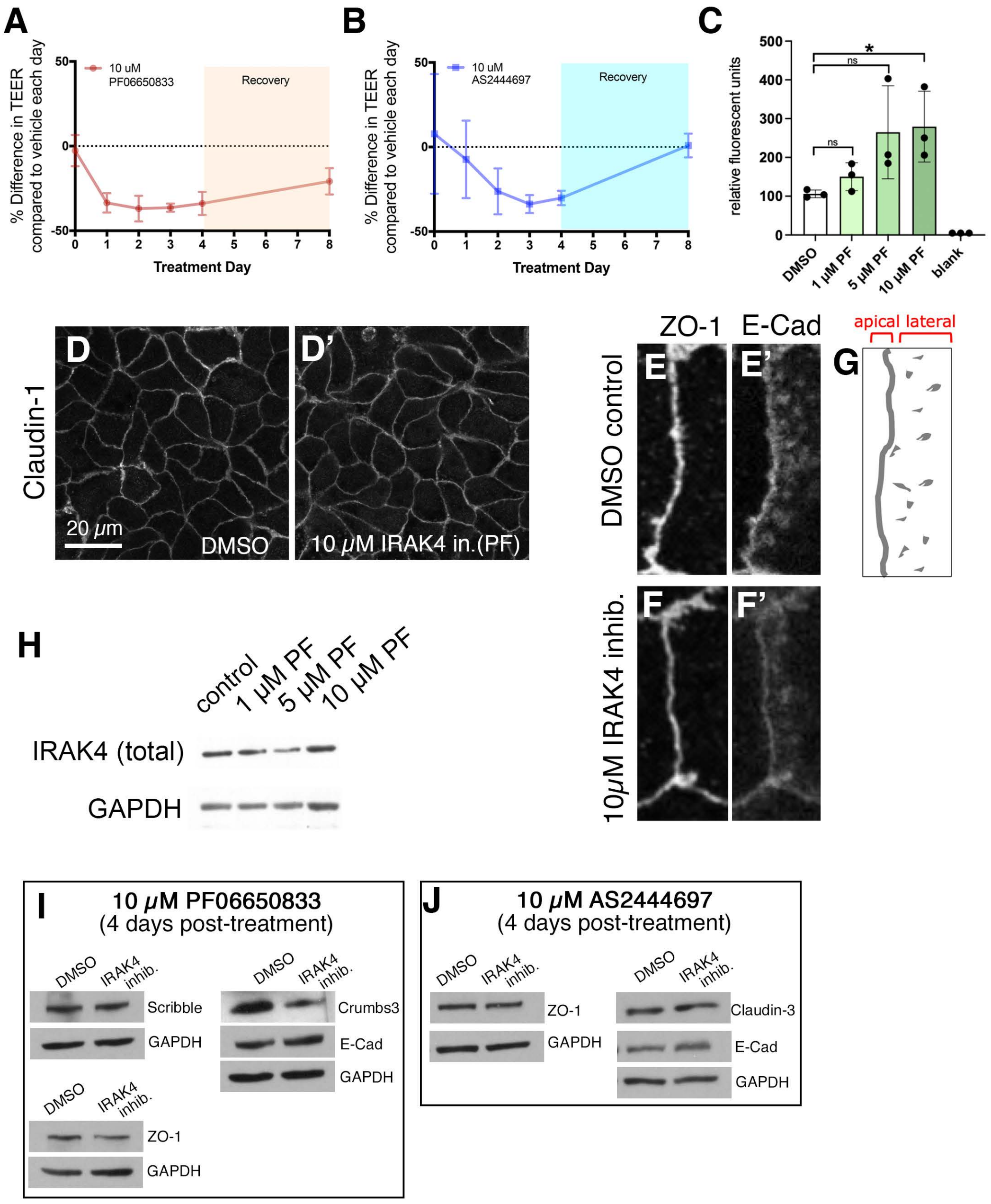

Supplemental Figure S2. Peterson et al.

Fig. S2, related to Figure 2. IRAK4 inhibition affects tight junction integrity and barrier function.

Treatment of 3-week post-confluent Caco-2 cells with 10μM of either PF06650888 (**A**) or AS 2444697 (**B**) IRAK4 inhibitor induces a dose-dependent reduction in TEER that is reversible upon inhibitor removal (recovery), indicating that the epithelial barrier at tight junctions is re-enforced upon washout of the inhibitor. Shown are SEM of n=3 separate transwells per experimental condition (DMSO, AS, PF). Statistical significance for the final timepoint was determined by unpaired Student's t-test. **C** Transepithelial diffusion of a fluorescent dextran FD4 across mature Caco-2 monolayers. Diffusion into the basal compartment is higher following 10μM PF-06650833 pre-treatment, suggesting diminished barrier integrity. Each data point represents an independent transwell (N=3). Statistical significance determined by one way ANOVA with Dunnett's multiple comparison test (* = $p<0.05$).

D-D' Effect of 10μM PF inhibitor-treatment on junctional components. Z-projections of confocal sections covering the apical-lateral junctional area are shown. The junctional intensity of Claudin-1 is decreased compared to control (DMSO)-treatment. Quantification is in Fig. 2H.

E-G E-Cadherin at apical junctions appears to change its distribution within lateral spot adherens junctions (see schematic in **G**) upon 10μM IRAK4 inhibitor-treatment (PF). ZO-1 (**E**, **F**) is shown in comparison to E-Cadherin (**E'**, **F'**) and to mark the apical-most end of the lateral sides.

H Treatment with IRAK4 inhibitor (PF) at different concentrations does not affect total IRAK4 levels. GAPDH is shown as loading control.

I, J Analysis of protein levels for control (DMSO)- and 10μM PF-treated (**I**) or 10μM AS-treated (**J**) Caco-2 cell monolayers. Immunoblots of samples revealed with the indicated antibodies are shown.

Development • Supplementary information

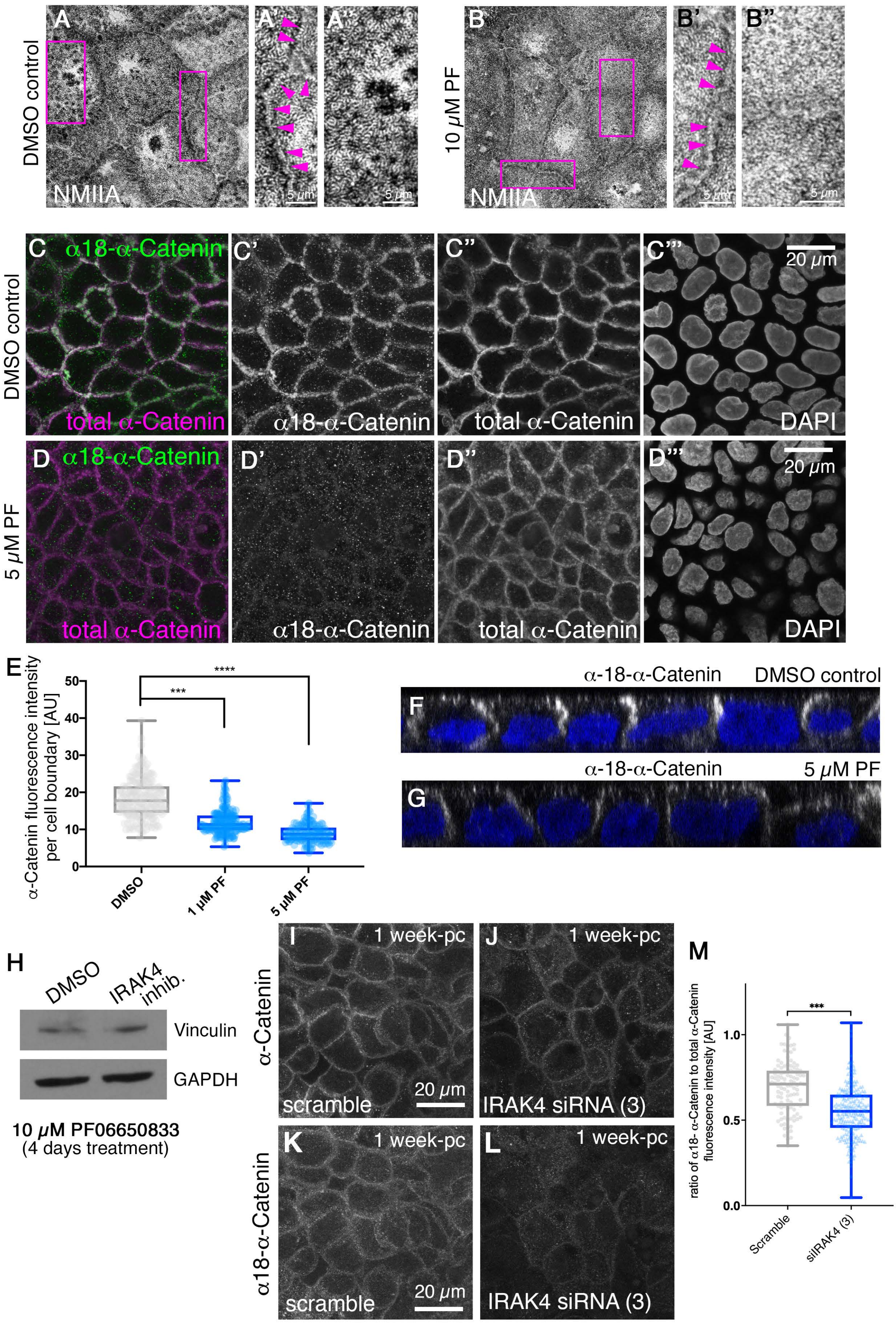

Supplemental Figure S3. Peterson et al.

Development • Supplementary information

Fig. S3, related to Figure 4. IRAK4 inhibition leads to loss of epithelial tension at tight junctions.

A-A'' In Caco-2 cells at 3-weeks-post confluence, NMIIA is organised into a prominent striated pattern at junctional contacts (**A'**, arrowheads) and across the apical surface (**A''**). Upon PF-inhibitor-treatment, NMIIA is less organised both at junctions (**B'**) as well as within the apical surface (**B''**). Magnified areas are indicated by magenta boxes. Panels **A** and **B** are also shown as two-colour panels in Fig. 4 B and D.

C-D''' Immunofluorescence of α18-α-Catenin compared to total α-Catenin (as quantified in Fig. 3K). Z-projections of confocal sections covering the apical-lateral junctional area are shown.

E Total α-Catenin fluorescence intensity at junctions in control and PF-inhibitor treated Caco-2 cells. Values are extracted from α-Catenin/α18-α-Catenin ratios (Figure 4K) and statistical significance was determined using one-way ANOVA with Dunnett's multiple comparison test as either p<0.0001 (***) or p<0.00001 (****).

F, G Cross-sections corresponding to panels **A'** and **B'** above illustrating α18-α-Catenin localisation at lateral junctions in control (DMSO) and 5µM PF-inhibitor treated Caco-2 cell monolayers.

H Vinculin total protein levels do not change between control and PF-inhibitor-treated Caco-2 cells, as analysed by Western blotting.

I-M In Caco-2 monolayers treated with siRNA(3) (see Figure 3) against IRAK4, both α-Catenin (**I**, **J**) as well as α18-α-Catenin (**K**, **L**) levels at junctions are reduced compared to control, with changes to α18-α-Catenin stronger than to total α-Catenin. Z-projections of confocal sections covering the apical-lateral junctional area are shown. **M** Quantification of α-Catenin/α18-α-Catenin ratio: n= 111 junctions were analysed for the scramble control, and n= 224 for the siRNA(3) against IRAK4. Statistical significance was determined using unpaired t-test as p<0.0001 (***).

Development • Supplementary information

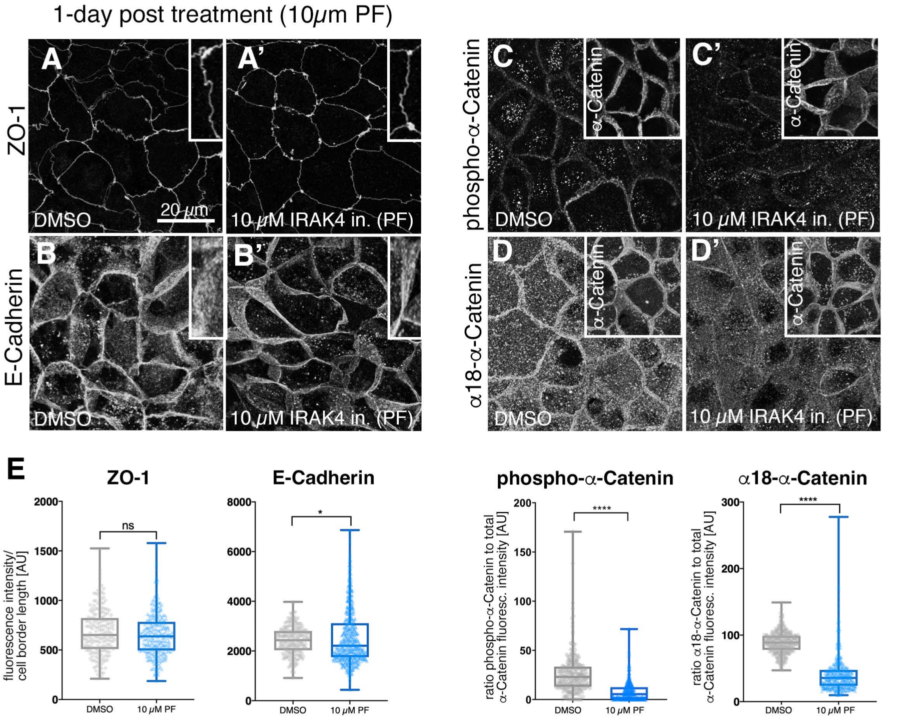

Suppl. Figure S4. Peterson et al.

Fig. S4, related to Figure 4. IRAK4 inhibition leads to changes at epithelial junctions one day post treatment.

A-D' Changes to junctional components upon treatment of 3-week post-confluence Caco2 monolayers at 1 day post treatment, showing DMSO-treated controls (**A**, **B**, **C**, **D**) and 10 μM PF treated cells (**A'**, **B'**, **C'**, **D'**), insets show individual junctions.

E Quantification of changes shown in **A-D'**. For ZO-1, n=200 for the vehicle control, n=344 for 10 μM PF treatment; for E-cadherin, n=269 for the vehicle control, n=669 for 10 μM PF treatment; for phospho-α-Catenin to total α-Catenin ratio, n=244 for the vehicle control, n=266 for 10 μM PF treatment; for a18-α-Catenin to total α-Catenin ratio, n=419 for the vehicle control, n=504 for 10 μM PF treatment. Statistical significance was determined using unpaired Student's t-test as either non significant (n.s.), $p<0.05$(*) or $p<0.0001$ (****).

Development • Supplementary information

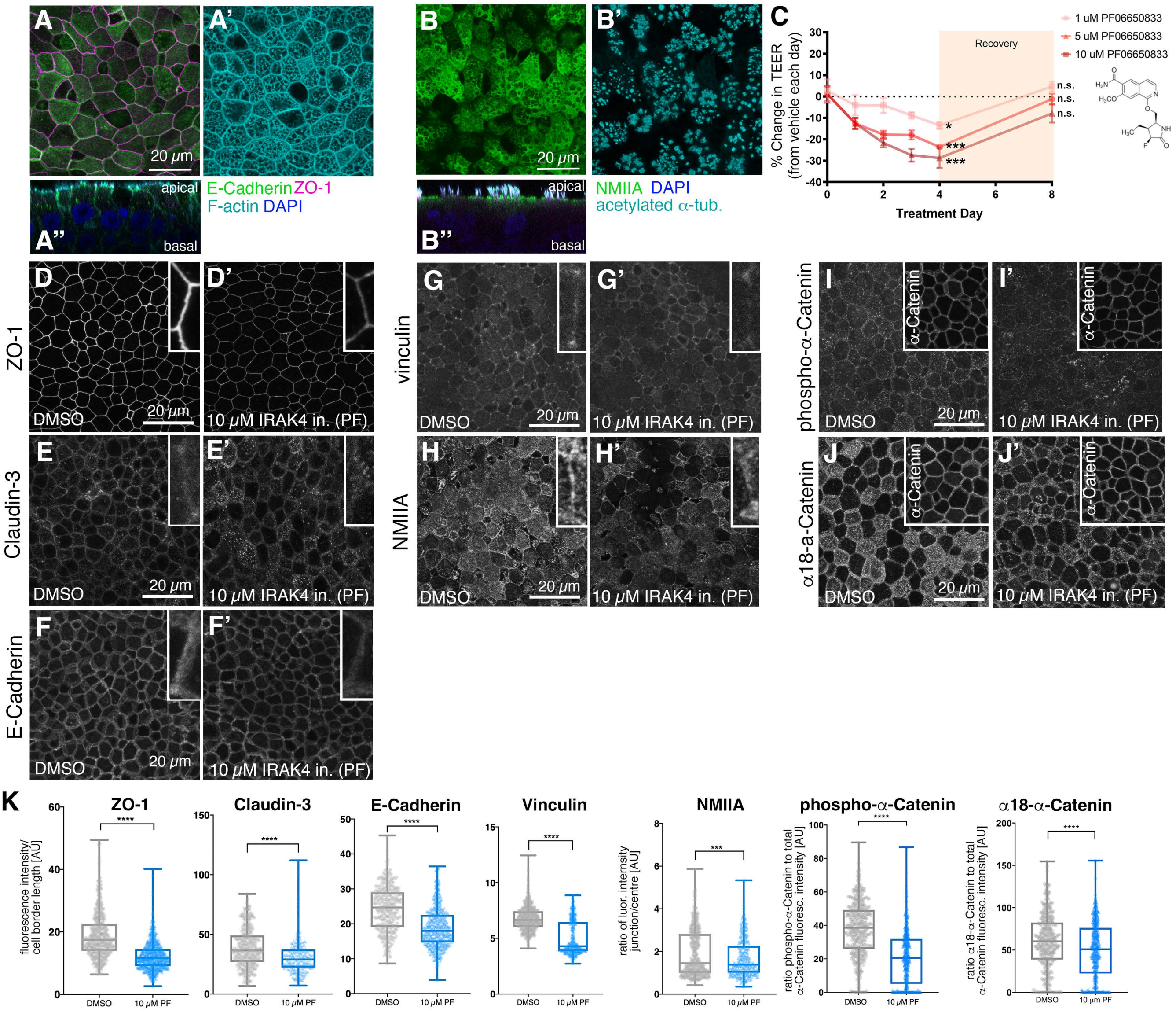

Suppl. Figure 5. Peterson et al.

Development • Supplementary information

Fig. S5, related to Figure 4. IRAK4 inhibition leads to loss of epithelial integrity and changes at epithelial junctions in primary bronchial respiratory cells. A-B'' Primary bronchial epithelial cells 2 weeks post-confluence show well-established tight and adherens junctions as well as an apical actomyosin web surrounding apical cilia. **A-A''** E-Cadherin (green), ZO-1 (magenta), F-actin (turquoise) and DAPI (blue) are shown, with **A''** showing a cross section. **B-B''** NMIIA (green), acetylated α-tubulin (turquoise) and DAPI (blue) are shown, with **B''** showing a cross section.

C Treatment of 2 week post-confluent primary bronchial cell monolayers with PF06650888 IRAK4 inhibitor induces a dose-dependent reduction in TEER that is reversible with 4 days of wash-out using inhibitor-free medium. Shown are SEM of n= 3 (DMSO), n=3 (PF) separate transwells. Statistical significance at day 4 and day 8 time point was determined by one-way ANOVA with Dunnett's multiple comparison test, with *<0.05, ** <0.005, ***<0.0005 and n.s. as non significant.

D-K Effect of PF inhibitor-treatment on junctional components. **D-J'** Z-projections of confocal sections covering the apical-lateral junctional area are shown for cells treated with PF-inhibitor, with quantification shown in **K**. ZO-1 (**D, D'**), Claudin-3 (**E, E'**) and E-Cadherin (**F, F'**), Vinculin (**G**, **G'**), NMIIA (**H**, **H'**), phospho-α-Catenin (**I**, **I'**) and α18-α-Catenin (**J**, **J'**) localisation at cell-cell junctions is reduced upon IRAK4 inhibition with 10μM PF. phospho-α-Catenin and α18-α-Catenin are shown and quantified in comparison to total α-Catenin (insets in **I-J'**). Insets in **D-H** show individual junctions.

K Quantification of fluorescence intensity changes at cell-cell junctions in control (DMSO), and 10μM PF treated primary bronchial cell layers. Total junctions analysed from 3 separate samples, one representative image per sample; for ZO1, n=535 for vehicle control, n=850 for 10μM PF; for Claudin-3, n=456 for vehicle control, n=255 for 10μM PF; for E-Cadherin, n=459 for vehicle control, n=650 for 10μM PF; for vinculin, n=580 for vehicle control, n=517 for 10μM PF; for NMIIA expressed as a ratio of junctional to central fluorescence, n=587 for vehicle control, n=473 for 10μM PF; for phospho-α-Catenin to total α-Catenin ratio, n=471 for the vehicle control, n=432 for 10 μM PF treatment; for a18-α-Catenin to total α-Catenin ratio, n=432 for the vehicle control, n=436 for 10 μM PF. Box-and-whisker plots in this and all subsequent figures show mean, 25th and 75th percentile, with extreme data points indicated by whiskers. Statistical significance was determined by unpaired t-test with Welch's correction or one-way ANOVA with Dunnett's multiple comparison test when comparing two or more drug treatments (**** = p<0.00001; *** = p<0.0001; * = p<0.01).

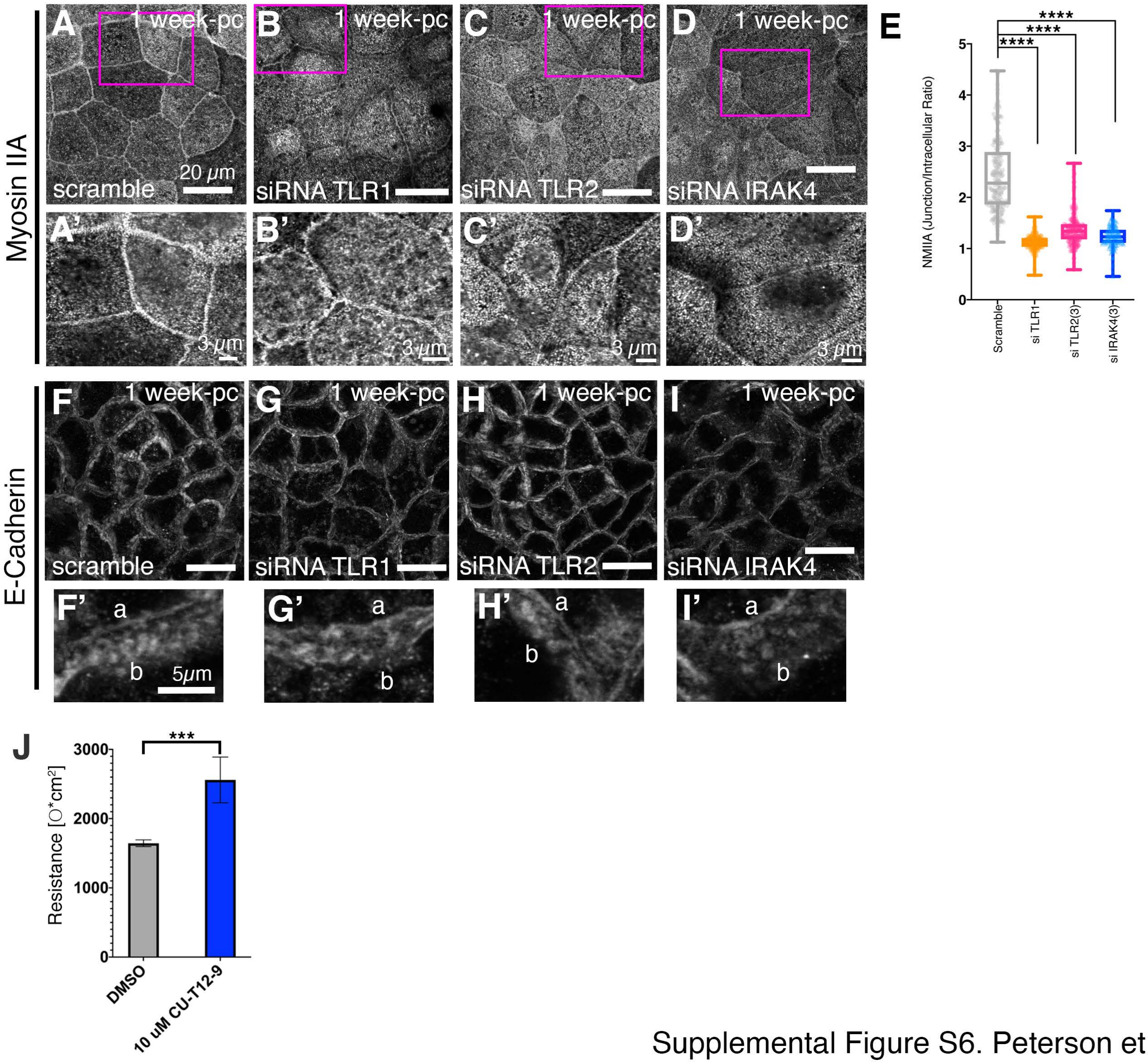

Supplemental Figure S6. Peterson et al.

Fig. S6, related to Figure 5. Reduction of TLR levels leads to loss of epithelial tightness and changes at junctions.

In siRNA-treated 1-week post-confluence Caco-2 monolayers with reduced TLR1 or TLR2 levels, localisation of junctional components to cell borders is affected. Z-projections of confocal sections covering the apical-lateral junctional area are shown.

A-E Knock-down of TLR1, TLR2 and IRAK4 leads to MNIIA reduction at junctions. **A-D** show overviews of the apical and junctional area of a field of Caco-2 cells, with **A'-D'** showing higher magnifications at the level of junctions, illustrating the reduction in junctional striations upon siRNA treatment. Magenta boxes indicate the position of the magnified panels.

Development • Supplementary information

E Quantification of effects on NMIIA: n= 182 junctions were analysed for the scramble control and n= 142 for siTLR1 and n=187 for siTLR2 and n= 151 for siIRAK4. Statistical significance was determined by one way ANOVA with Dunnett's multiple comparison test as **** = $p<0.00001$.

F-I' Knock-down of TLR1, TLR2 and IRAK4 leads to changes to E-Cadherin distribution at lateral junctions, similar to IRAK4-inhibitor treatment. **F'-I'** show higher magnifications of individual tilted lateral sides of Caco-2 cells, 'a' indicates the apical and 'b' the basal end of the lateral membranes shown.

J Treatment of Caco-2 cells with 10µM of the TLR1/2 agonist CU-T12-9 leads to an increase in TEER after 5 days in culture (upon seeding at confluency). Error bars indicate SD of n= 3 (DMSO, CU-T12-9) separate transwells. Statistical significance was determined by unpaired Student's t-test as *** = $p<0.0001$.

Table S1. Antibodies

Epitope	Vendor	Catalogue # or reference	Species	Fixative	IF [ ]	WB [ ]
Claudin 1	Thermofisher	51-9000	Rb Polyclonal	2% PFA	1:25	1:250
Claudin 3	Thermofisher	34-1700	Rb Polyclonal	2% PFA	1:25	1:250
Claudin 5	Thermofisher	34-1600	Rb Polyclonal	2% PFA	1:25	1:250
Crumbs3	Abcam	ab180835	Rt monoclonal	n/a	n/a	1:250
E-cadherin	BD Bioscience	610181	Ms Monoclonal	4% PFA	1:200	1:2500
GAPDH	Thermofisher	MA5-15738	Ms Monoclonal	4% PFA	n/a	1:5000
GFP	Abcam	ab290	Rb	4% PFA	1:200	
IRAK4	Abcam	ab32511	Rb Polyclonal	n/a	n/a	1:1000
Myd88	R&D	MAP3109	Ms Monoclonal	4% PFA		1:100
MyoIIa	Biolegend	909801	Rb Polyclonal	4% PFA		1:200
NfkB p65	CST	8242	Rb Monoclonal	4% PFA	1:1000	1:400
Occludin	Thermofisher	71-1500	Rb Polyclonal	2% PFA	1:50	1:50
p-IRAK4	CST	11927S	Rb Polyclonal	4% PFA		1:150
p-α-catenin, (S655/T658)	CST	13231	Rb Polyclonal	n/a	n/a	1:1000
Phalloidin 647	Thermofisher	A22284	n/a	4% PFA		1:500
Scribble	Santacruz	sc-11049	Gt	4% PFA	n/a	1:250
TLR1	Santacruz	sc-514399	Ms Monoclonal	n/a	n/a	1:250
TLR2	Abcam	ab16894	Ms Monoclonal	4% PFA	1:50	1:250
TLR4	Santacruz	sc-293072	Ms Monoclonal	4% PFA	1:50	1:250
TLR6	Proteintech	22240-1-AP	Rb Polyclonal	n/a	n/a	1:500
vinculin	SigmaAldrich	v9131	Ms Monoclonal	4% PFA	1:50	1:250
vinculin	Proteintech	66305	Rb Polyclonal	4% PFA	1:100	n/a
ZO-1	Thermofisher	61-7300	Rb Polyclonal	4% PFA	1:200	1:1000
ZO-1	Thermofisher	33-9100	Ms Monoclonal	4% PFA	1:200	1:1000

Development • Supplementary information

α-catenin	Santacruz	sc9988	Ms Monoclonal	4% PFA	1:200	1:1000
α18 a-catenin	Non-commercial	(Yonemura et al., 2010)	Rt monoclonal	4% PFA	1:200	

Note, for anti TLR antibodies, the antibodies used in our study were the best performing in IF and WB of many that are available that we assessed.

Development • Supplementary information
